# The causal relationship between smoking and thoracic aortic aneurysm: Evidence from Mendelian randomization analysis

**DOI:** 10.1097/MD.0000000000038361

**Published:** 2024-05-31

**Authors:** Jianwei Zhou, Yafeng Wang

**Affiliations:** a Department of Cardiology Xishuangbanna Dai Autonomous Prefecture People's Hospital No. 4, Jinghong, Yunnan.

**Keywords:** Mendelian randomization, smoking, thoracic aortic aneurysm

## Abstract

The potential role of smoking as a risk factor for thoracic aortic aneurysm is still a subject of debate. Therefore, it is important to systematically investigate the causal relationship between smoking and thoracic aortic aneurysm using Mendelian randomization methods. Genetic data were obtained from genome-wide association studies using the inverse variance weighting method as the primary approach. A thorough sensitivity analysis was conducted to ensure the reliability of the findings. Instrumental variables were assessed using the *F* statistic, and meta-analysis was employed to assess the average genetic predictive effect between smoking and thoracic aortic aneurysm. Our Mendelian randomization study found a positive association between smoking and thoracic aortic aneurysm. The odds ratios (OR) in the inverse variance weighting method were OR = 1.23 (95% confidence interval [CI] = 1.00–1.51; *P* = .053) and OR = 2.07 (95% CI = 1.10–3.91; *P* = .024). Furthermore, meta-analyses consistently demonstrated a positive causal relationship between ferritin and myocardial infarction, although statistical significance was not observed. The analysis results did not indicate any horizontal pleiotropy. Despite the presence of heterogeneity, the Mendelian randomization analysis still yielded significant results. This study employed Mendelian randomization to establish a positive association between smoking levels and the risk of thoracic aortic aneurysm. The genetic evidence reveals a causal relationship between the two, offering new insights for future interventions targeting thoracic aortic aneurysms.

## 1. Introduction

Aortic aneurysms are defined as significant dilations of the aorta exceeding normal diameter by 50% or surpassing 3 cm.^[1]^ A rupture of an aneurysm can precipitate severe internal hemorrhaging, often resulting in fatality; indeed, mortality rates posthospitalization reach 80%, and postoperative death occurs in 50% of surgical interventions for aortic aneurysm rupture.^[2,3]^ In 2017, aortic aneurysms accounted for 167,200 fatalities and the loss of 3 million disability-adjusted life years, as reported by the Global Burden of Disease Study.^[4]^ Aortic aneurysms are categorized based on the location of dilation within the aorta, with classifications including abdominal aortic aneurysm (AAA) and thoracic aortic aneurysm (TAA).^[5]^ The risk factors and determinants of AAA have undergone rigorous investigation, yielding numerous insights into the pathogenesis and potential strategies for intervention. A meta-analytical survey by Altobelli et al^[6]^ identified gender, smoking habits, hypertension, coronary artery disease, and familial history as significant contributors to AAA risk, findings consistent across populations. Similarly, a narrative review by Teixeira et al^[7]^ substantiated the overlap of several risk factors between AAA and atherosclerotic disease. The correlation between smoking and the risk of AAA has been well-established by previous meta-analyses.^[8]^ Conversely, observational studies exploring the association between smoking and TAA are limited, leaving a gap in understanding this potential link. To address this, we aim to elucidate the causal relationship between smoking and TAA through a Mendelian randomization (MR) study.

MR offers a novel approach for elucidating causal relationships between disease entities. It capitalizes on genome-wide association studies (GWAS) data to identify specific nucleotide variations known as single-nucleotide polymorphisms (SNPs). These genetic variations, acting as instrumental variables (IVs), emulate the random distribution of diseases, akin to the natural assortment in gamete formation. MR employs these variants as proxies for exposures, smoking behavior for instance, untainted by confounding factors like diet, alcohol intake, or socioeconomic status. Traditional study designs such as cohort or case-control studies may fall short due to complications like reverse causation, where disease potentially influences smoking habits, and residual confounding. MR’s interpretive strength lies in its reliance on the random allocation of alleles during fertilization, mirroring the randomization in controlled trials. Existing MR research has confirmed a significant causal link between smoking and the development of aortic aneurysms.^[9]^ Moreover, smoking has been implicated as a risk factor for AAAs.^[10]^ However, the causal relationship between smoking and TAAs remains to be clarified.

## 2. Materials and methods

### 2.1. Study design

**Figure 1.**
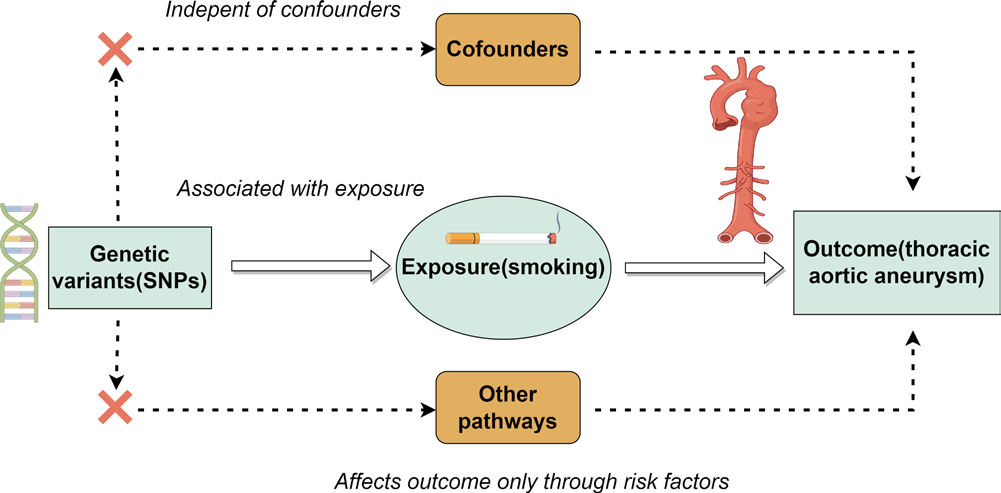
The causal relationship between smoking and thoracic aortic aneurysm was explored by MR. The concept and 3 core assumptions of MR analysis are shown in the figure above. We created the figure with “www.figdraw.com” By Figdraw. MR = Mendelian randomization.

In this study, MR studies must fulfill the 3 essential assumptions of correlation, independence, and exclusion, that is: the IV must exhibit a strong correlation with the exposure factor; the IV must not display any correlation with any confounding factor that is connected to the exposure-result association; and the IV can solely impact the outcome variable via the exposure factor (Fig. 1). We extracted consolidated information from openly accessible databases (OpenGWAS, FinnGen) which had received informed consent from the participants and had been granted ethical approval.

The smoking data used in this study were sourced from 2 different GWAS datasets with the identifiers ieu-b-25 and ukb-b-10831, respectively. The TAA data, on the other hand, were obtained from the GWAS dataset with the identifier finngen_R10_I9_THAORTANEUR. The smoking data were retrieved from the IEU Open GWAS database (https://gwas.mrcieu.ac.uk/), while the TAA data were obtained from the FinnGen database (https://www.finngen.fi/en/access_results). Further details and summary information can be found in Table 1.

**Table 1:** Characteristics of data used in the Mendelian randomization study.

Exposure/outcomes	GWAS ID	Ethnicity	Sample size	Number of SNPs	Source
Cigarettes per day	ieu-b-25	European	337,334	11,913,712	IEU
Pack years of smoking	ukb-b-10831	European	142,387	9,851,867	IEU
Thoracic aortic aneurysm	finngen_R10_I9_THAORTANEUR	European	385,857	19,345,258	FinnGen

GWAS = genome-wide association study, SNPs = single-nucleotide polymorphisms.

### 2.2. IV selection criteria

We selected independent genetic variants that displayed genome-wide significance (*P* < 5 × 10^−8^), specifically SNPs, from the exposed dataset. To ensure the independence of these SNPs, we eliminated any linkage disequilibrium (correlation coefficient *r*^2^ < 0.001) within a genetic distance of kb = 10,000. Following this criterion, we identified the exposed IVs by intersecting these SNPs with the ending SNPs and removing any palindromic SNPs with intermediate gene frequencies. We calculated the *F* value statistic of the IV to evaluate its strength. If the *F* value statistic of IV is >10, then it is considered a strong IV; otherwise, it is considered a weak IV.

### 2.3. MR analysis

For this research, the examination of the association between smoking and outcomes of TAA was conducted using various approaches, namely inverse variance weighting (IVW),^[11]^ weighted median,^[12]^ weighted mode,^[13]^ and MR-Egger methods.^[14]^ IVW was used as the gold standard if Cochrane *Q* test (*P* < .05), and if *P* > .05, the IVW fixed model was used as the gold standard.^[15]^

### 2.4. Sensitivity analysis

To assess the reliability of the findings from the MR research, we conducted a sensitivity analysis. We employed Cochran *Q* test to identify any discrepancies in the data, with a significance level of *P* < .05 indicating the existence of heterogeneity. For the detection of horizontal pleiotropy and outliers, we utilized the MR-Pleiotropy RESidual Sum and Outlier (PRESSO) technique. In case any outliers were detected, they were eliminated, and an outlier-corrected MR analysis was conducted to obtain an impartial causal estimation. To evaluate the presence of directional pleiotropy, we employed the MR-Egger-intercept test. We analyzed whether the presence of a single SNP significantly affected the MR results by using a leave-one-out test. The R package TwoSampleMR (version 0.5.6) was utilized for the MR analysis, while the detection of MR-PRESSO was carried out using the R package MR-PRESSO in R program (version 4.3.2).

### 2.5. Meat-analysis

In this investigation, we conducted a meta-analysis utilizing 2 GWAS datasets related to smoking in order to genetically anticipate smoking’s impact. The “meta” package in R software (version 4.3.2) was utilized to conduct the meta-analysis. To visually evaluate the pooling outcomes, we created Forrest plots. The level of heterogeneity among the studies was estimated using *I*^2^ and the Chi-squared-based *Q*.

## 3. Results

**Figure 2.**
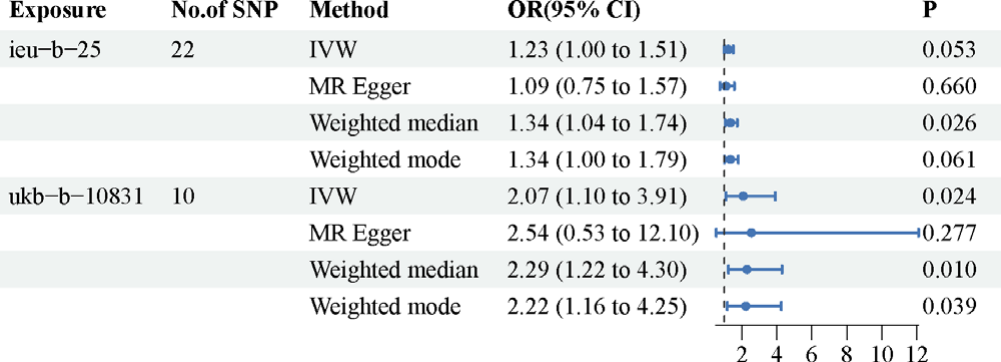
Forest plot of the causal relationship between smoking and thoracic aortic aneurysm. Smoking may be a risk factor for the incidence of thoracic aortic aneurysm. CI = confidence interval, IVW = inverse variance-weighted, MR = Mendelian randomization, OR = odds ratio.

**Figure 3.**
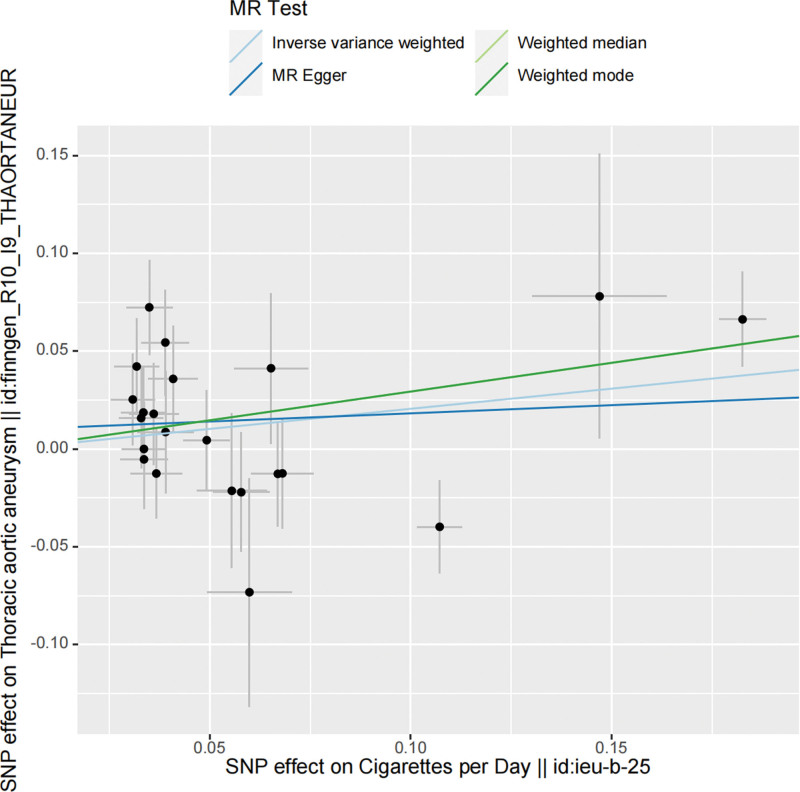
Scatter plot showing the causal relationship between smoking and thoracic aortic aneurysm (exposure number: ieu-b-25). MR = Mendelian randomization, SNP =  single-nucleotide polymorphism.

**Figure 4.**
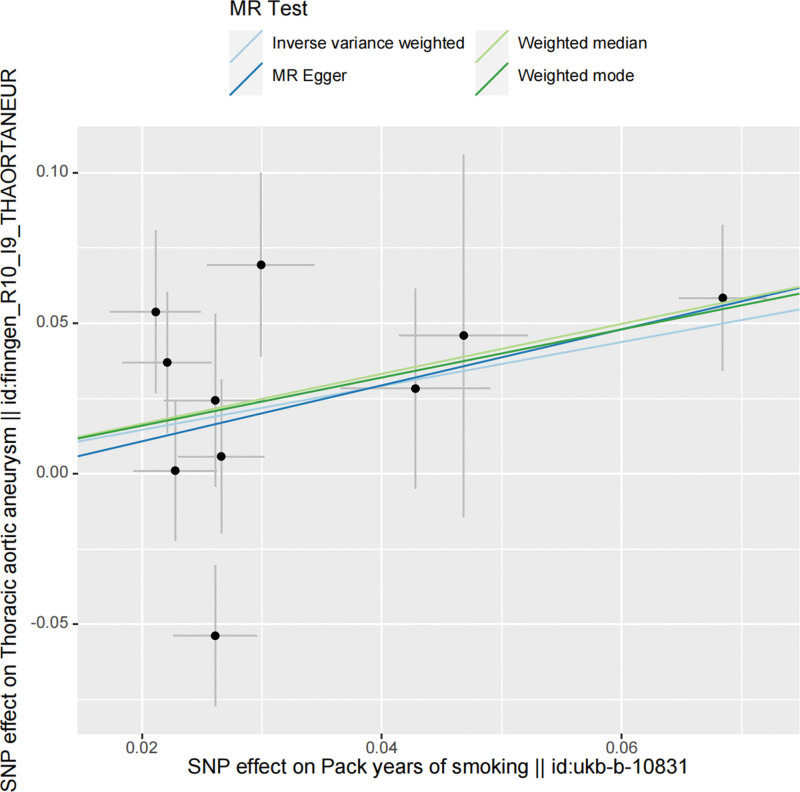
Scatter plot showing the causal relationship between smoking and thoracic aortic aneurysm (exposure number: ukb-b-10831). MR = Mendelian randomization, SNP =  single-nucleotide polymorphism.

Two different GWAS datasets were utilized to enhance the credibility of MR studies on smoking. The *F* statistics for all SNPs in our dataset were >10, indicating the absence of weak IVs. Regarding the causal relationship between smoking and TAA, the first analysis (Exposure ID: ieu-b-25) revealed a potential positive association, but it lacked statistical significance. However, in the analysis of the second dataset (Exposure ID: ukb-b-10831), a significant and evident positive correlation was observed. IVW results showed odds ratios of OR = 1.23 (95% confidence interval [CI] = 1.00–1.51; *P* = .053) and OR = 2.07 (95% CI = 1.10–3.91; *P* = .024) for Mendelian smoking and TAA, respectively. The results of the randomization analysis method are presented in Figure 2, while scatter plots are depicted in Figures 3 and 4.

**Figure 5.**
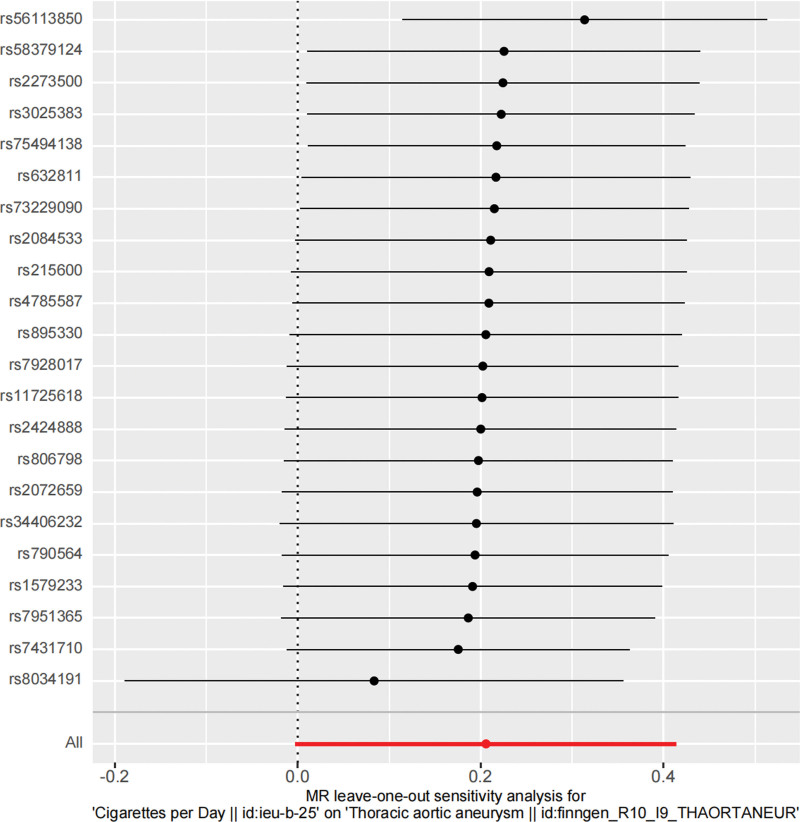
Leave-one-out plot of SNPs related to smoking and thoracic aortic aneurysm (exposure number: ieu-b-2). MR = Mendelian randomization, SNP =  single-nucleotide polymorphism.

**Figure 6.**
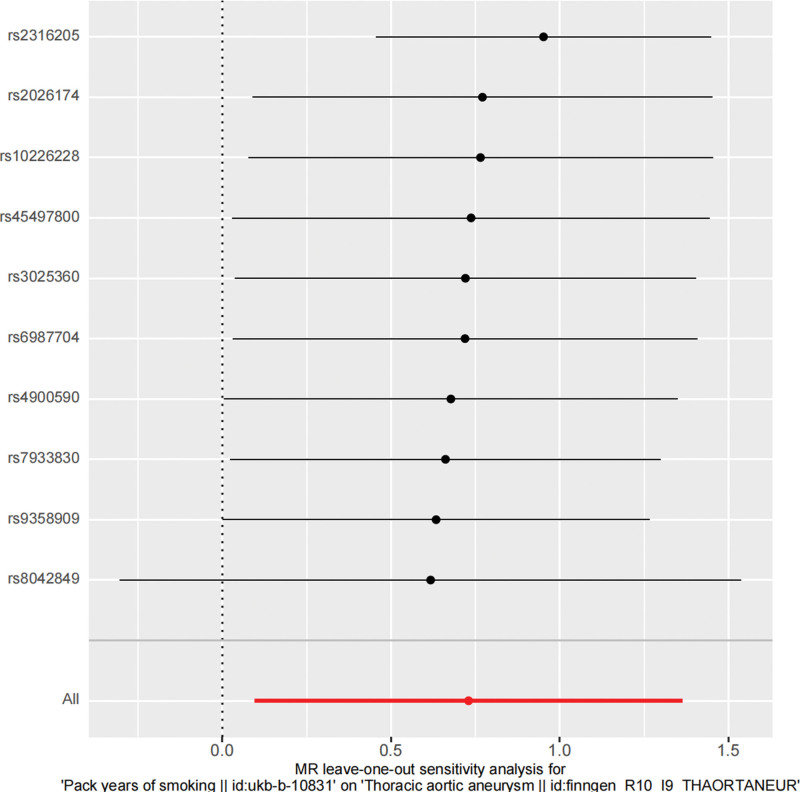
Smoking and thoracic aortic aneurysm (exposure number: ukb-b-10831) Leave-one-out plot of related SNPs. MR = Mendelian randomization, SNP =  single-nucleotide polymorphism.

To assess the heterogeneity of the data, Cochrane *Q* test (IVW and MR-Egger methods) was used. The *P* value of the IVW method in the first set of analysis was .074 (*P* > .05), the *P* value of MR-Egger was .079 (*P* > .05), and the *P* value of the IVW method in the second set of analysis was .047 (*P* < .05). It can be seen that there is no heterogeneity in the first group. Although there is heterogeneity in the second group, the IVW results in MR analysis are still reliable. Egger in the 2 groups The *P* values obtained by the intercept method are all >0.05, indicating that TAA is not affected by IVs other than smoking. MR-STRO was used to confirm the absence of horizontal pleiotropy and outliers in the 2 datasets (*P* = .096 and *P* = .132 > .05). In addition, leave-one-out testing demonstrated the stability of the results, as shown in Figures 5 and 6.

**Figure 7.**
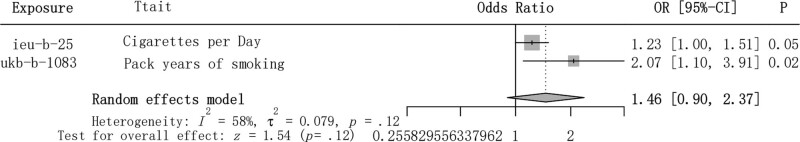
Meta-analysis forest plot of 2 different smoking datasets. CI =  confidence interval.

To obtain stronger evidence, a meta-analysis was performed on 2 datasets and the *I*^2^ test was used to assess heterogeneity among other datasets. The results show that there is heterogeneity between the 2 datasets (*I*^2^ = 58% > 50%). Therefore, a random effects model was adopted, and the results showed that there was a positive association between smoking and TAA, but it was not statistically significant (OR = 1.46, 95% CI = 0.90–2.37, *P* = .12), as shown in Figure 7.

## 4. Discussion

The association of smoking with TAA exhibits significant variability across different age groups and sexes, influenced by a complex interplay of genetic, behavioral, and physiological factors. Smoking is a well-established risk factor for TAA, with its impact being more pronounced in certain demographics. Studies have shown that male sex is a potent nonmodifiable risk factor for TAA, with men having a 4- to 10-fold higher incidence than women. This disparity is attributed to biological sex and gender differences affecting cardiovascular disease susceptibility and outcomes, including TAA.^[16]^ However, the risk associated with smoking is substantial across both sexes. Women, particularly those who smoke, have been found to be more sensitive to the development of TAAs, suggesting that smoking exacerbates the risk of TAA more significantly in women than in men.^[17]^ Age also plays a critical role in the association between smoking and TAA. Older individuals, especially those who have a history of smoking, are at a higher risk.^[18]^ This is consistent with global trends showing an age-dependent increase in the burden of aortic aneurysms, although the age-standardized rate of death from these conditions has been decreasing, partly due to reductions in smoking rates.^[19]^ Interestingly, the impact of smoking on TAA risk is not uniform across all smokers. The duration of smoking cessation has been shown to influence the risk, with a more rapid decline in excess risk observed among women compared to men after quitting smoking.^[20,21]^ This suggests that smoking cessation is particularly beneficial in reducing TAA risk, highlighting the importance of targeted public health interventions. Furthermore, the interaction between smoking, sex, and other risk factors such as body mass index and hypertension indicates a complex relationship. For instance, a higher body mass index has been associated with increased mortality from aortic disease among men and smokers, but this association is not straightforward and varies by sex and smoking status.^[22,23]^ In summary, the association of smoking with TAAs is influenced by sex and age, with men and older individuals being at higher risk. However, women who smoke are particularly sensitive to developing TAAs, underscoring the need for gender-specific interventions and smoking cessation programs to mitigate this risk.^[24,25]^

Prior research has robustly associated smoking with the incidence and progression of aortic aneurysms. Wilmington TBM’s investigation into the impact of smoking, duration of smoking, and cessation on AAA risk uncovered that smokers have a 7.6-fold increased likelihood of developing aneurysms relative to nonsmokers, with former smokers facing a tripling of risk.^[26]^ Annually, smoking elevates the risk of AAA rupture by 4% (95% CI = 2%), designating long-term smoking as a preeminent environmental risk factor for AAA development.^[27]^ Contrastingly, research on the connection between smoking and TAA is scant. One retrospective study reported no significant correlation.^[28]^ Whereas a prospective cohort study suggested that smoking may precipitate TAA.^[29]^ MR studies have previously established causality between smoking, AAA, and intracranial aneurysm.^[10,30]^ Our current analysis posits a potential causal link with TAA, although meta-analytic data did not achieve statistical significance. The meta-analysis did not reveal a statistically significant association between the 2 factors. This outcome may be influenced by several variables, including limitations in sample size, heterogeneity in study design, and potential confounders, all of which could attenuate the statistical power of the tests. Particularly in observational studies, it is challenging to completely eliminate all potential confounding factors, such as lifestyle habits that may be associated with both smoking and TAAs.

The exact mechanisms by which smoking promotes aortic aneurysm development remain to be fully elucidated. Initial studies linked smoking to enhanced aortic elastase activity, based on rabbit models.^[31]^ Buckley’s subsequent research implicated smoking in the induction of inflammatory mediators, inciting a sustained inflammatory state within arterial walls that promotes matrix metalloproteinases activation and the degradation of elastic fibers, thereby exacerbating aneurysm dilation.^[32]^ Another hypothesis posits that smoking may augment the effects of angiotensin II in aneurysm formation.^[33]^ Zhao et al suggest the 5-lipoxygenase pathway’s pivotal role in hyperlipidemia-associated aortic aneurysm pathogenesis.^[34]^ Although the pathogenesis is complex, smoking’s significant impact as an environmental factor on aortic aneurysms’ development is undeniable, warranting further exploration.

This study harnesses MR’s strengths: the analysis of causal relationships via genetic data avoids issues of reverse causation and confounding; it uses genetic variation as an IV, providing insights akin to randomized controlled trials in contexts where such trials are impractical. Furthermore, the large sample sizes and robust association with SNPs enhance the precision of causal inference. Nonetheless, the study is not without limitations. The predominance of European samples may limit the generalizability of our findings, and the absence of detailed subgroup data precludes analyses across different genders or age brackets concerning smoking’s causal relationship with TAA.

## 5. Conclusions

In summary, while conventional meta-analyses have not demonstrated a statistically significant correlation between smoking and TAAs, MR analysis of genetic data suggests a potential causal link. This genetic-based analysis underscores the importance of carefully considering the risks of smoking in the development of disease prevention strategies. Furthermore, it provides a fresh perspective for future research, emphasizing the significance of understanding disease from a genetic standpoint.

## Acknowledgments

The authors express gratitude to OpenGWAS and FinnGen for openly providing these aggregated data.

## Author contributions

**Conceptualization:** Jianwei Zhou.

**Data curation:** Jianwei Zhou.

**Formal analysis:** Jianwei Zhou, Yafeng Wang.

**Funding acquisition:** Jianwei Zhou.

**Investigation:** Jianwei Zhou.

**Methodology:** Jianwei Zhou.

**Project administration:** Jianwei Zhou.

**Resources:** Jianwei Zhou.

**Software:** Jianwei Zhou.

**Supervision:** Jianwei Zhou.

**Validation:** Jianwei Zhou.

**Visualization:** Jianwei Zhou.

**Writing—original draft:** Jianwei Zhou.

**Writing—review & editing:** Jianwei Zhou, Yafeng Wang.

## References

[1] Hu J Li Y Wei Z. A reduction in the vascular smooth muscle cell focal adhesion component syndecan-4 is associated with abdominal aortic aneurysm formation. Clin Transl Med. 2021;11:e605. 34936241 10.1002/ctm2.605 PMC8693440

[2] Verhoeven EL Kapma MR Groen H. Mortality of ruptured abdominal aortic aneurysm treated with open or endovascular repair. J Vasc Surg. 2008;48:1396–400. 18829222 10.1016/j.jvs.2008.07.054

[3] Basnyat P Biffin A Moseley L. Mortality from ruptured abdominal aortic aneurysm in Wales. Br J Surg. 1999;86:765–70. 10383576 10.1046/j.1365-2168.1999.01170.x

[4] Aune D Sen A Kobeissi E Hamer M Norat T Riboli E. Physical activity and the risk of abdominal aortic aneurysm: a systematic review and meta-analysis of prospective studies. Sci Rep. 2020;10:22287. 33339835 10.1038/s41598-020-76306-9 PMC7749100

[5] Isselbacher EM. Thoracic and abdominal aortic aneurysms. Circulation. 2005;111:816–28. 15710776 10.1161/01.CIR.0000154569.08857.7A

[6] Altobelli E Rapacchietta L Profeta VF Fagnano R. Risk factors for abdominal aortic aneurysm in population-based studies: a systematic review and meta-analysis. Int J Environ Res Public Health. 2018;15:2805. 30544688 10.3390/ijerph15122805 PMC6313801

[7] Teixeira FG Silva V Batista P. Abdominal aortic aneurysm: risk factors, new diagnosis realities and complications prevision. J Cardiol Curr Res. 2015;3:7–7.Teixeira FG， Silva V， Batista P.腹主动脉瘤：危险因素、新诊断现实和并发症预想。 J Cardiol Curr Res. 2015;3:7–7.

[8] Aune D Schlesinger S Norat T Riboli E. Tobacco smoking and the risk of abdominal aortic aneurysm: a systematic review and meta-analysis of prospective studies. Sci Rep. 2018;8:14786. 30283044 10.1038/s41598-018-32100-2 PMC6170425

[9] Zhou J Lin J Zheng Y. Association of cardiovascular risk factors and lifestyle behaviors with aortic aneurysm: a Mendelian randomization study. Front Genet. 2022;13:925874. 36003339 10.3389/fgene.2022.925874 PMC9393757

[10] Larsson SC Mason AM Bäck M. Million Veteran Program. Genetic predisposition to smoking in relation to 14 cardiovascular diseases. Eur Heart J. 2020;41:3304–10. 32300774 10.1093/eurheartj/ehaa193 PMC7544540

[11] Burgess S Butterworth A Thompson SG. Mendelian randomization analysis with multiple genetic variants using summarized data. Genet Epidemiol. 2013;37:658–65. 24114802 10.1002/gepi.21758 PMC4377079

[12] Bowden J Davey Smith G Haycock PC Burgess S. Consistent estimation in Mendelian randomization with some invalid instruments using a weighted median estimator. Genet Epidemiol. 2016;40:304–14. 27061298 10.1002/gepi.21965 PMC4849733

[13] Hartwig FP Davey Smith G Bowden J. Robust inference in summary data Mendelian randomization via the zero modal pleiotropy assumption. Int J Epidemiol. 2017;46:1985–98. 29040600 10.1093/ije/dyx102 PMC5837715

[14] Bowden J Davey Smith G Burgess S. Mendelian randomization with invalid instruments: effect estimation and bias detection through Egger regression. Int J Epidemiol. 2015;44:512–25. 26050253 10.1093/ije/dyv080 PMC4469799

[15] Bowden J Hemani G Davey Smith G. Invited commentary: detecting individual and global horizontal pleiotropy in Mendelian randomization—a job for the humble heterogeneity statistic? Am J Epidemiol. 2018;187:2681–5. 30188969 10.1093/aje/kwy185 PMC6269239

[16] Yang L Murugesan A Martin S Colvard B Janko M. Abdominal aortic aneurysm prevalence in smoking-related cancers and sex differences. J Am Coll Surg. 2022;235:S313–4.

[17] Wang Z You Y Yin Z. Burden of aortic aneurysm and its attributable risk factors from 1990 to 2019: an analysis of the global burden of disease study 2019. Front Cardiovasc Med. 2022;9:901225. 35711350 10.3389/fcvm.2022.901225 PMC9197430

[18] Takada M Yamagishi K Tamakoshi A Iso H; JACC Study Group. Body mass index and mortality from aortic aneurysm and dissection. J Atheroscler Thromb. 2021;28:338–48. 32727971 10.5551/jat.57232 PMC8147012

[19] Prakash SK Milewicz DM. X marks the spot: the profound impact of sex on aortic disease. Am Heart Assoc. 2018;38:9–11. 10.1161/ATVBAHA.117.310433 PMC5751955 29282246

[20] Hozawa A. Screening for aortic aneurysm: further evidence is required to clarify the issue. J Atheroscler Thromb. 2021;28:319–319. 32981920 10.5551/jat.ED143 PMC8147017

[21] Sharples L Sastry P Freeman C. Aneurysm growth, survival, and quality of life in untreated thoracic aortic aneurysms: the effective treatments for thoracic aortic aneurysms study. Eur Heart J. 2022;43:2356–69. 34849716 10.1093/eurheartj/ehab784 PMC9246658

[22] Jahangir E Lipworth L Edwards TL. Smoking, sex, risk factors and abdominal aortic aneurysms: a prospective study of 18 782 persons aged above 65 years in the Southern Community Cohort Study. J Epidemiol Community Health. 2015;69:481–8. 25563744 10.1136/jech-2014-204920 PMC4494088

[23] Stackelberg O Björck M Larsson S. Sex differences in the association between smoking and abdominal aortic aneurysm. J Br Surg. 2014;60:1394–5. 10.1002/bjs.9526 24916023

[24] Yang Y Yamagishi K Kihara T. Smoking cessation and mortality from aortic dissection and aneurysm: findings from the Japan Collaborative Cohort (JACC) study. J Atheroscler Thromb. 2023;30:348–63. 35718450 10.5551/jat.63258 PMC10067343

[25] Nicolini F Vezzani A Corradi F. Gender differences in outcomes after aortic aneurysm surgery should foster further research to improve screening and prevention programmes. Eur J Prev Cardiol. 2018;25(1_suppl):32–41. 29708035 10.1177/2047487318759121

[26] Wilmink TB Quick CR Day NE. The association between cigarette smoking and abdominal aortic aneurysms. J Vasc Surg. 1999;30:1099–105. 10587395 10.1016/s0741-5214(99)70049-2

[27] Forsdahl SH Singh K Solberg S Jacobsen BK. Risk factors for abdominal aortic aneurysms: a 7-year prospective study: the Tromsø Study, 1994–2001. Circulation. 2009;119:2202–8. 19364978 10.1161/CIRCULATIONAHA.108.817619

[28] Kim JB Kim K Lindsay ME. Risk of rupture or dissection in descending thoracic aortic aneurysm. Circulation. 2015;132:1620–9. 26338955 10.1161/CIRCULATIONAHA.114.015177

[29] Landenhed M Engström G Gottsäter A. Risk profiles for aortic dissection and ruptured or surgically treated aneurysms: a prospective cohort study. J Am Heart Assoc. 2015;4:e001513. 25609416 10.1161/JAHA.114.001513 PMC4330075

[30] Zeng C Huang Z Tao W. Genetically predicted tobacco consumption and risk of intracranial aneurysm: a Mendelian randomization study. Environ Sci Pollut Res Int. 2023;30:12979–87. 36117221 10.1007/s11356-022-23074-w

[31] Cohen JR Sarfati I Wise L. The effect of cigarette smoking on rabbit aortic elastase activity. J Vasc Surg. 1989;9:580–2. 2709526

[32] Buckley C Wyble CW Borhani M. Accelerated enlargement of experimental abdominal aortic aneurysms in a mouse model of chronic cigarette smoke exposure. J Am Coll Surg. 2004;199:896–903. 15555973 10.1016/j.jamcollsurg.2004.08.010

[33] Stolle K Berges A Lietz M Lebrun S Wallerath T. Cigarette smoke enhances abdominal aortic aneurysm formation in angiotensin II-treated apolipoprotein E-deficient mice. Toxicol Lett. 2010;199:403–9. 20937366 10.1016/j.toxlet.2010.10.005

[34] Zhao L Moos MP Gräbner R. The 5-lipoxygenase pathway promotes pathogenesis of hyperlipidemia-dependent aortic aneurysm. Nat Med. 2004;10:966–73. 15322539 10.1038/nm1099

